# Supplementary material for: Modulation of Allicin-Free Garlic on Gut Microbiome
Source: Molecules. 2020 Feb 5;25(3):682. doi: 10.3390/molecules25030682 (PMC7036987; doi:10.3390/molecules25030682)
Supplement: Supplementary file 1 [file molecules-25-00682-s001.zip › molecules-693324-SM/Table S1Nutrient analysis.pdf]

Appendix Table S1. Nutrient analysis 2017

Nutrients	AFG	Method
**Calorie (kcal/100g)**	357.5	Calorie=Protein*4+Lipid*9+Carbohydrates*4
**Protein (g/100g)**	13.05	Kaida's method
**Lipid (g/100g)**	1.4	Acid decomposition method
**Carbohydrates (g/100g)**	73.2	100-(protein+lipid+moisture+ash)
**Salt (g/100g)**	0.02	Sodium conversion
**Sodium (mg/100g)**	8.5	Atomic absorption spectroscopy
**Moisture (g/100g)**	8.9	High pressure drying
**Ash (g/100g)**	3.75	Ashing method
**Fructan (mg/g)**	468.8	Fructan analysis kit
**G-SAC (mg/g)**	23.675	HPLC
**Alliin (mg/g)**	17.415	HPLC
**SAC (mg/g)**	0.785	HPLC
**Allicin (mg/g)**	0	HPLC
**Organosulfide (mg/g)**	41.875	
**Fructan/Sulfide**	11.195	
