# Supplementary material for: Modulation of Allicin-Free Garlic on Gut Microbiome
Source: Molecules. 2020 Feb 5;25(3):682. doi: 10.3390/molecules25030682 (PMC7036987; doi:10.3390/molecules25030682)
Supplement: Supplementary file 1 [file molecules-25-00682-s001.zip › molecules-693324-SM/Table S2 Dietary.pdf]

Appendix Table S2. Dietary compositions of each group

Components (%)	ND	ND 1AFG	ND 5AFG	HFD	HFD 1AFG	HFD 5AFG
Lard	6.0	6.0	6.0	40.0	40.0	39.8
Corn starch	54.0	54.0	49.0	20.0	20.0	15.2
Casein	21.0	21.0	21.0	21.0	21.0	21.0
Sucrose	10.0	9.0	10.0	10.0	9.0	10.0
Cellulose	4.0	4.0	4.0	4.0	4.0	4.0
Mineral mix	3.5	3.5	3.5	3.5	3.5	3.5
Vitamin mix	1.0	1.0	1.0	1.0	1.0	1.0
Choline chloride	0.2	0.2	0.2	0.2	0.2	0.2
Methionine	0.3	0.3	0.3	0.3	0.3	0.3
GS (Garlic)	——	1.0	5.0	——	1.0	5
Total calories (kcal/100g)	367.8	365.1	369.1	567.4	564.7	567.6
